# Intracavernosal metaraminol bitartrate for treatment of priapism resulting from circumcision: a case report

**DOI:** 10.1186/s40064-016-2069-9

**Published:** 2016-04-12

**Authors:** Min Tang, Bianjiang Liu, Jie Li, Qiang Lu, Ninghong Song, Zengjun Wang, Wei Zhang

**Affiliations:** State Key Laboratory of Reproductive Medicine, Department of Urology, The First Affiliated Hospital of Nanjing Medical University, 300 Guangzhou Road, Nanjing, 210029 People’s Republic of China

**Keywords:** Circumcision, Injection, Intracavernosal, Metaraminol, Priapism

## Abstract

**Introduction:**

Priapism is an uncommon disorder of involuntary prolonged erection beyond sexual excitement or desire. Herein, we present a rare case of priapism resulting from traditional circumcision under regional anesthesia with dorsal penile nerve block by xylocaine, which was successfully treated by intracavernosal injection of metaraminol bitartrate.

**Case description:**

A 37-year-old man visited our out-patient department for a penile erection, which had been observed during the surgery, lasting for 21 days. 10 days after circumcision, he accepted simple corporeal aspiration in another hospital but it had no effect. In our hospital, he was injected intracavernosally twice a day, with 2 mg metaraminol bitartrate diluted in 1 ml normal saline every time. Complete resolution of penile tumescence was achieved after injection for 7 days, no complications were observed.

**Discussion and evaluation:**

Priapism developed following circumcision is very uncommon. This particular case was diagnosed as high-flow non-ischemic priapism, and is the first reported event of priapism resulting from circumcision which was finally successfully treated with the efficient and minimally invasive method of intracavernosal injection of metaraminol bitartrate.

**Conclusions:**

Intracavernous injection of metaramino bitartrate might be a simple, effective and safe method for relief of priapism associated with circumcision. Yet, more clinical studies are needed to validate the effectiveness of intracavernosal metaramino bitartrate for post-circumcision priapism.

## Introduction

Priapism is an uncommon disorder of involuntary prolonged erection beyond sexual excitement or desire, usually accompanied by penile pain. Early and effective treatment could avoid long-term erectile dysfunction (ED). The most common causes were trauma (Koga et al. 1990), intracavernosal pharmacotherapy for ED, the hyperviscosity syndromes, psychotropic medications, malignant infiltration (Keoghane et al. 2002), spinal or general anaesthesia (Van Arsdalen et al. 1983; Roy 1984) and so on. Nevertheless, the mechanisms have not been fully understood.

Herein, we report a rare case of priapism result from traditional circumcision that was successfully treated by intracavernosal injection of metaraminol bitartrate.

## Case description

**Fig. 1:**
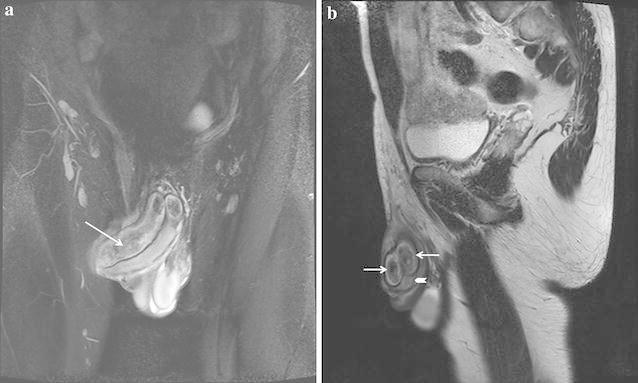
Magnetic resonance imaging of the erected penis. MRI (Heavily T2-weighted image) shows both sides of corpus cavernosum (*arrow*) increased significant thickly, as well as the corpus spongiosum (*arrowhead*). **a** Coronal view, **b** sagittal view

An otherwise healthy 37-year-old man was admitted on April 2, 2015 complaining of an erection, lasting for 21 days. He had accepted traditional circumcision under regional anesthesia with dorsal penile nerve block by xylocaine 21 days ago. Penile erection was noticed during the operation, but was not paid enough attention to. Nevertheless, Priapism was persistent and did not respond to simple corporeal aspiration 10 days after development of the priapism, but without discomfort of dysuria or pain. He then came to our out-patient department. On physical examination the corpora cavernosa were erect and the corpus spongiosum was flaccid. He had received ultrasonography in another hospital where he accepted corporeal aspiration, but uncarefully missed the report. Rapidly, we got the cavernosal blood gas analysis, and the result showed pH, 7.437; carbon dioxide pressure, 42.5 mmHg; oxygen pressure, 88.5 mmHg; and oxygen saturation, 97.5 %. Further MRI showed both sides of corpus cavernosum increased significant thickly, as well as the corpus spongiosum (Fig. 1). Based on the history, clinical manifestation and examination results, we then made the diagnosis of high flow non-ischaemic priapism in this case.

He was then injected intracavernosally at one side of the penile draft twice a day over 7 days, with 2 mg metaraminol bitartrate diluted in 1 ml normal saline every time. A total of 28 mg was given. Some softening of the penis was noticed at the first injection although it became hard again 30 min later. The second injection resulted in moderate detumescence. The penis achieved complete flaccid after the fourteenth injection and remained soft. Blood pressure and heart rate which were measured immediately before and 3 min after metaraminol injection, and there were no significant changes (not shown). Follow-up for 2 months showed that no recurrent penile tumescence was observed.

## Discussion and evaluation

Priapism is a rare condition defined as a persistent penile erection arising from dysfunction of the mechanisms regulating penile tumescence and flaccidity, and the etiology is still unclear.

Priapism developed following circumcision is even more uncommon. As far as we know, there have been only two reports about priapism associated with circumcision till now. Canter and Coskuner (2011) presented a case that priapism developed 2 days after circumcision but did not respond to decompression attempts with pharmacologic treatment, and glandulocavernous shunt was needed in the first 12 h after development of the priapism. But severe penile necrosis developed almost 3 weeks after circumcision and priapism. Another case of long-term priapism that caused by circumcision, complicated local skin necrosis, was treated with traditional Chinese therapy, such as bone scraping and depletion therapy, and achieved satisfactory effect (Jin et al. 2005). Both of the above two cases were not without drawbacks, result in either ischemia event or further-term ED. To the best of our knowledge, this particular case is the first reported event of priapism resulting from circumcision which was finally successfully treated with an efficient and minimally invasive method, intracavernosal injection of metaraminol bitartrate.

It is widely accepted that there are two different types of priapism. One is low-flow ischemic priapism which is caused by stagnation of blood in the corpus cavernosum, the other is high-flow non-ischemic which is caused by an excess of arterial inflow relative to venous outflow (Koga et al. 1990). There might be a limitation in this case that we did not reexamine ultrasonography when the patient arrived at our department. But the role of cavernosal blood gas analysis in typing diagnosis of priapism has been generally confirmed by clinical practice. The blood gas result was normal in this case. Moreover, except for the noneffective corporeal aspiration, the patient did not accept any other treatment before coming to our department. Thus the erected penis had formed mild corporal fibrosis when he arrived, according to the MRI result. Taken together, our case was considered to be high-flow non-ischemic priapism. As the therapy of high-flow priapism is not as urgent as that of low-flow priapism because there is no risk of ischemia, conservative therapeutic and close observation is recommended by most urologists (Pautler and Brock 2001). Thus we chose intracavernosal treatment considering the minimal trauma.

Brindley (1984) was the first one to report that intracavernosal injection of metaraminol causes shrinkage of the penis. He described that the alpha-adrenergic receptor existed in the cavernous smooth muscle and penile arteries. Intracorporeal instillation of metaraminol, which is an alpha-adrenergic agent, contracts penile arteries and the arterial flow in the corpus cavernosum decrease, and thus effective to priapism. This method was then widely practiced in impotent patients who develop priapism as a complication of pharmacologically induced penile erection (Brindley 1986). Then, Tsai and Hong found that intracavernosal injection of 10–25 pg metaraminol is a simple, effective and safe method for immediate relief of intraoperative penile erection. It can be used in patients with either regional block or general anaesthesia (Tsai and Hong 1990). Koga et al. (1990) firstly reported that a 30-year-old male with post-traumatic priapism for 7 days was treated successfully by metaraminol bitartrate injection into the corpus cavernosum. In that case, metaramino bitartrate 2–4 mg (20 % solution) was injected into the corpus cavernosum six times over 10 days, and a total dose of 19 mg was given. Treatment of priapism using metaraminol had been suggested in the hospital setting as described above. Interestingly, Mcdonald firstly reported successful management of recurrent priapism secondary to sickle cell trait using self-administered injections of intracavernosal metaraminol at home, with the frequency as often as once daily using 5–10 mg of drug (McDonald and Santucci 2004).

Through analysis of the literature data and patient’s general situation, we chose 2 mg metaramino bitartrate as the initial dose. The blood pressure and heart rate were monitored immediately before and 3 min after injection, and no changes were observed. Since starting injection, he did not report any systemic side effects associated with sympathomimetics such as headache, shortness of breath, flushing or chest pain. Some decrease of rigidity was observed 5 min after injection, but the penis became elastic-hard again 30 min later. We then injected as often as twice a day, 2 mg metaramino bitartrate each time. Complete resolution of penile tumescence was achieved after injection for 7 days. After 2 months of follow-up, no recurrent priapism is found, and the erectile function is general normal.

## Conclusions

We concluded that intracavernous injection of metaramino bitartrate might be a simple, effective and safe method for relief of priapism associated with circumcision. It should be tried at first before any more invasive methods, such as corporeal aspiration or shunting techniques. The first few times of injections may not be so effective, but our case shows that repeat injection several times may be still effective. Yet, more clinical studies are needed to gather experience about the category of priapism and corresponding prescriptions of metaramino bitartrate.

## Abbreviations

ED: erectile dysfunction
MRI: magnetic resonance imaging
